# Sedation improves early outcome in severely septic Sprague Dawley rats

**DOI:** 10.1186/cc8012

**Published:** 2009-08-19

**Authors:** Hong Qiao, Robert D Sanders, Daqing Ma, Xinmin Wu, Mervyn Maze

**Affiliations:** 1 Department of Anesthesiology, First Hospital, Peking University, No. 8 Xishiku St., Beijing 100034, PR China; 2 Department of Anaesthetics, Intensive Care and Pain Medicine, Imperial College London, Chelsea & Westminster Hospital, 369 Fulham Rd, London, SW10 9NH, UK

## Abstract

**Introduction:**

Sepsis, a systemic inflammatory response to infective etiologies, has a high mortality rate that is linked both to excess cytokine activity and apoptosis of critical immune cells. Dexmedetomidine has recently been shown to improve outcome in a septic cohort of patients when compared to patients randomized to a benzodiazepine-based sedative regimen. We sought to compare the effects of dexmedetomidine and midazolam, at equi-sedative doses, on inflammation and apoptosis in an animal model of severe sepsis.

**Methods:**

After central venous access, Sprague Dawley rats underwent cecal ligation and intestinal puncture (CLIP) with an 18 G needle without antibiotic cover and received either saline, or an infusion of comparable volume of saline containing midazolam (0.6 mg.kg-1.h-1) or dexmedetomidine (5 ug.kg-1.h-1) for 8 hours. Following baseline measurements and CLIP, blood was sampled for cytokine measurement (tumour necrosis factor (TNF)-alpha and interleukin (IL)-6; n = 4-6 per group) at 2, 4 and 5 hours, and animal mortality rate (MR) was monitored (n = 10 per group) every 2 hours until 2 hours had elapsed. In addition, spleens were harvested and apoptosis was assessed by immunoblotting (n = 4 per group).

**Results:**

The 24 hour MR in CLIP animals (90%) was significantly reduced by sedative doses of either dexmedetomidine (MR = 20%) or midazolam (MR = 30%). While both sedatives reduced systemic levels of the inflammatory cytokine TNF-alpha (*P *< 0.05); only dexmedetomidine reduced the IL-6 response to CLIP, though this narrowly missed achieving significance (*P *= 0.05). Dexmedetomidine reduced splenic caspase-3 expression (*P *< 0.05), a marker of apoptosis, when compared to either midazolam or saline.

**Conclusions:**

Sedation with midazolam and dexmedetomidine both improve outcome in polymicrobial severely septic rats. Possible benefits conveyed by one sedative regimen over another may become evident over a more prolonged time-course as both IL-6 and apoptosis were reduced by dexmedetomidine but not midazolam. Further studies are required to evaluate this hypothesis.

## Introduction

Sepsis affects 750,000 patients per year in the USA, killing 250,000 of these people. In the UK severe sepsis has a mortality rate of 45% [1-3] and despite putative therapeutic options including early goal-directed therapy [4] and activated protein C [5], outcome in septic patients has not vastly improved. Septic pathogenesis involves multiple mechanisms including inflammation, organ malperfusion and apoptosis of critical cells including lymphocytes and enterocytes [2,3]. The inflammatory response is initially exaggerated (best exemplified in meningoccemia or toxic-shock syndrome) at which stage anti-inflammatory therapy may have some utility [6]. Following this phase of injury a hypo-inflammatory phase ensues that is characterized by the apoptosis of B and T lymphocytes and subsequent failure of the adaptive and innate immune systems [2,3].

Sedative agents exert anti-inflammatory effects that may differentially effect this biphasic inflammatory response to sepsis. Initially, their anti-inflammatory effects may prove beneficial by reducing the 'cytokine storm'; in this case early institution of sedation may contribute to the benefits of early goal-directed therapy. Indeed, anti-inflammatory agents in early, severe sepsis [7-10] or those with high circulating IL-6 levels [8,11] may prove useful. Equally plausible, the sedative-induced anti-inflammatory effect may exacerbate the subsequent immunosuppression in the secondary hypo-inflammatory phase and potentiate lymphocyte apoptosis [12]. Sedatives affect immune responses directly [13,14] but may also modulate these processes by indirect mechanisms such as through the burden of sleep deprivation [15] and effects on autonomic nervous system activity [16,17].

Accumulating evidence suggests that the currently used sedatives may exert a deleterious effect in the presence of infection [14], notably morphine and benzodiazepines increase mortality from bacterial infections in animals [18-20]. Clinical epidemiological evidence also suggests an association between chronic benzodiazepine usage and increased severity of community-acquired pneumonia [21]. In contrast, dexmedetomidine improves mortality from endotoxic shock in rats [22] and cecal ligation and intestinal puncture in mice [23] associated with an anti-inflammatory effect. Clinically, the anti-inflammatory effects of dexmedetomidine have proven superior to both midazolam [24] and propofol [25]. In addition, dexmedetomidine has organ-protective effects and can inhibit apoptotic cell death [26] that plays a pivotal role in the pathogenesis of sepsis [2,3]. Stimulation of α_2 _adrenoceptors also enhances the phagocytic ability of macrophages *in vitro *[27-29] and thus may enhance bacterial clearance by the innate immune system. The sympatholytic effects of α_2 _adrenoceptor agonists may be useful as sympatholysis has been shown to improve outcome in septic animals [30]. Finally, dexmedetomidine induces a sedative state more analogous to natural sleep than benzodiazepines and therefore we hypothesize that dexmedetomidine could reduce immune dysfunction related to sleep deprivation [31]. Recently we performed a secondary analysis of data from the MENDS trial [32] revealing a mortality benefit in septic patients sedated with dexmedetomidine relative to lorazepam. In order to understand whether this represents an advantage of dexmedetomidine or a deleterious effect of the benzodiazepine we have utilised a model of acute severe sepsis to understand whether the choice of sedative influences outcome in the early phase where hyper-inflammation is an important contributor to mortality.

## Materials and methods

The study protocol conforms with the United Kingdom Animals (Scientific Procedures) Act of 1986, the Home Office (UK) and was approved by the local institutional review board.

Sixty 10 to 14 week old, male Sprague-Dawley rats weighing 340 to 390 g were used in this study. Animals were acclimatized to laboratory conditions for three days before experimental use, housed at 21°C with a 12-hour light-dark cycle, and allowed free access to tap water and standard rodent chow. On the day of study, the rats were weighed and anesthetized with an intraperitoneal injection of pentobarbital sodium 50 mg/kg repeated twice (every three hours). The internal jugular vein was cannulated to draw blood samples and for the sedative infusion. The rats were then randomized to saline infusion (C group), midazolam infusion at 0.6 mg/kg/hr (M group) or dexmedetomidine infusion at 5 μg/kg/hr (D group) [22] for eight hours (n = 20 per group) with equal volume infusion rate at 1 ml/hr in each group. Drug doses were calculated from human doses scaled for body surface area using the Meeh-Rubner formula. The dexmedetomidine dose had previously been applied in rats [22] and the midazolam dose was the calculated equivalent of the dexmedetomidine dose for the rat (scaled from human dosing). All animals appeared sedated and did not need further sedation to maintain immobility. All groups were administered intravenous fluids at 1 ml/hr (thus ensuring the same volume of fluid resuscitation). After this procedure, the animals were rested for 30 minutes followed by a baseline venous blood sample (time = 0 hours). Body temperature was maintained at 37 ± 0.2°C with the aid of a heating pad.

### Cecal ligation and double intestinal puncture

After cannulation and the start of the sedative infusions cecal ligation and double intestinal puncture (CLIP) was performed as previously described [33,34] under additional pentobarbital anesthesia. The procedure was performed under sterile conditions with the abdominal skin disinfected with 70% alcohol. Laparotomy was conducted through a 2 cm lower-midline incision. The cecum was exposed and ligated immediately distal to the ileocecal valve to avoid intestinal obstruction and then punctured twice with an 18-gauge needle, squeezed gently to force out a small amount of feces, and then returned to the abdominal cavity. The abdomen is closed with 3-0 silk sutures in two layers. Following completion of CLIP the sedative (or saline) infusions were continued without bolus administration.

### Plasma cytokine measurement

Venous blood samples (1 ml) were drawn for the measurement of plasma cytokine (TNF-α and IL-6) concentrations at two, four, and six hours after CLIP (n = four to six per group). Double the volume of saline was injected to replace blood lost after each sampling. A total amount of 4 mL of blood was drawn from each animal over eight hours. Samples were centrifuged at 3500 rpm for 10 minutes at 4°C and plasma was collected and stored frozen at -80°C until assaying. IL-6 and TNF-α were measured in duplicate using a commercially available ELISA kit (Biosource, CA, USA). The sensitivities of the assays were 3 pg/ml for IL-6 and 3 pg/ml for TNF-α and 3 pg/ml for IL-6.

### Western blot methodology

At the end of the experimental period (nine hours for western blot experiments) spleens were harvested (n = four per group). The samples of spleen were then homogenized (Polytron homogenizer by Kinematica, Bethlehem, PA, USA) in ice-cooled lysis buffer (20 mm Tris-HCl, 150 mm NaCl, 1 mm Na_2_DTA, 1 mm EGTA, 1% Triton, 2.5 mm sodium pyrophosphate, 1 mm β-glycerophosphate, 1 mm Na_3_VO_4_, 2 mm dl-dithiothreitol, 1 mm phenylmethanesulfonyl, and 1 μg/ml leupeptin; pH 7.5) and centrifuged at 3000 *g *for 10 minutes at 4°C. The supernatant was further centrifuged twice, initially at 12,000 *g *for 15 minutes at 4°C and a second time at 20,000 *g *for 45 minutes at 4°C. The protein concentration of supernatant was determined with the Bradford protein assay (Bio-Rad, Herts, UK). The supernatant (10 μg protein per sample) were denaturated in NuPAGE LDS Sample buffer (Invitrogen, Paisley, UK) at 70°C for 10 minutes and then were loaded on a NuPAGE 4 to 12% Bis-Tris Gel (Invitrogen, Paisley, UK). After electrophoresis, the proteins were electrotransferred to a nitrocellulose membrane (Hybond ECL; Amersham Biosciences, Buckinghamshire, UK) and incubated with a blocking solution composed of 5% fat dry milk in Tween-containing Tris-buffered saline (pH 8.0, 10 mm Tris, 150 mm NaCl, 0.1% Tween). The blocked membrane was incubated overnight at 4°C with the cleaved caspase-3 antibody (New England Biolab, Hitchin, United Kingdom). After washing with Tween-containing Tris-buffered saline for four times, the membrane was incubated for one hour at room temperature with the appropriate horseradish peroxidase-conjugated secondary antibody directed at the primary antibody. The bands were then visualized with enhanced chemiluminescence (New England Biolab, Hitchin, United Kingdom) and exposed onto Hyperfilm ECL film (Amersham Biosciences, Buckinghamshire, United Kingdom). Subsequently, the membrane was re-probed with caspase 3 and beta-action primary antibody respectively and the rest procedures were repeated again as above. The band density was analyzed densitometrically and normalized with the housekeeping protein beta-actin and then presented as percentage of control.

### Mortality rate

Animals were monitored every two hours via video recording of the animal in its cage following the initial eight-hour sedative infusion period and animal mortality was noted (n = 10 per group). After 16 hours of follow up (i.e., 24 hours post CLIP) all animals were sacrificed by lethal sodium pentobarbital injection.

### Statistics

The results are presented as mean ± standard error of the mean. Statistical analysis was performed by analysis of variance followed by *post-hoc *Newman Keuls testing using the instat program. Twenty-four hour mortality was analyzed by Chi squared test. A *P *< 0.05 was set as significant.

## Results

### Animal illness and mortality

**Figure 1:**
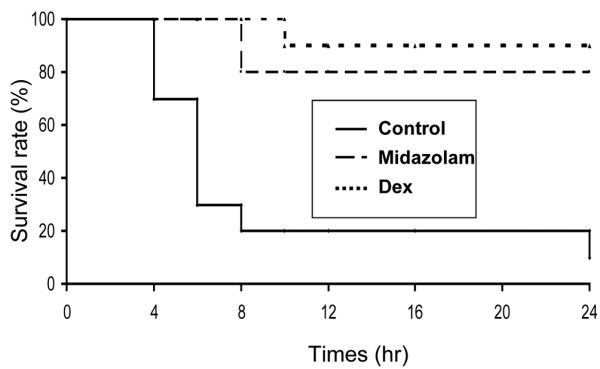
Kaplan-Meier survival curves for saline, midazolam or dexmedetomidine treated severely septic rats.  Dex = dexmedetomidine.

The CLIP model employed induced severe sepsis with lethargy and sickness behavior observable in the saline-infused animals. Nine of the 10 animals died within 24 hours (90%) indicating that very severe sepsis was provoked (Figure 1). Sedation with either drug significantly decreased mortality at 24 hours after CLIP compared with saline (*P *< 0.01; midazolam 30% and dexmedetomidine 20% mortality, respectively). However, no difference was noted between dexmedetomidine and midazolam (*P *= 0.6).

### Cytokine signalling: TNF-α

**Figure 2:**
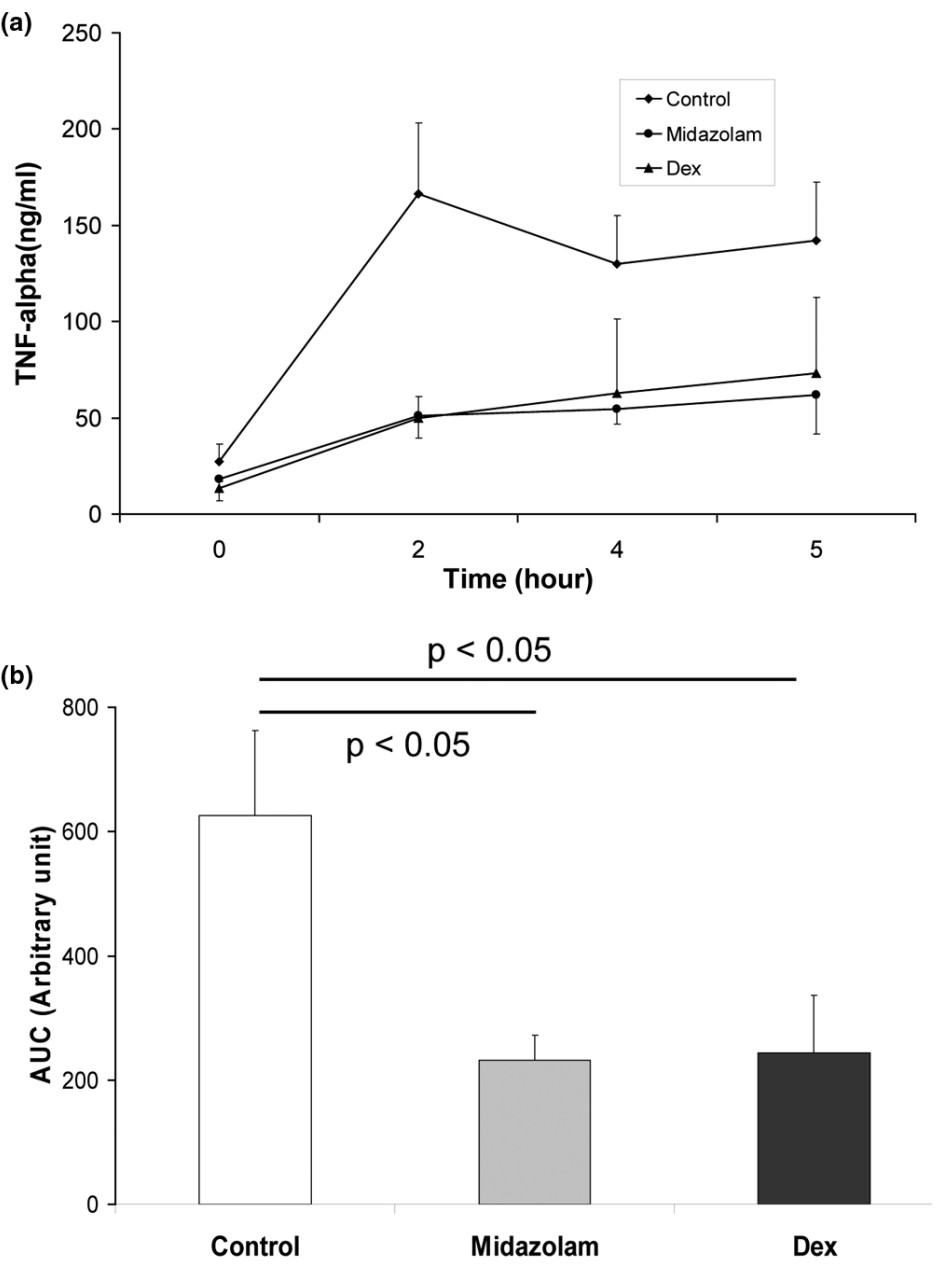
Plasma TNF-α levels immediately prior to (0 hours) and after (2, 4 and 5 hours) induction of severe sepsis by double caecal ligation and puncture in rats (n = 4 to 6).  **(a) **The actual change in levels is shown. **(b) **The total difference in levels via analysis of area under the curve (AUC) is shown. Dex = dexmedetomidine.

Both midazolam and dexmedetomidine reduced TNF-α levels compared with saline-treated controls. At two hours the saline group had a significantly higher level (166 ± 37 pg/ml) than either midazolam (51 ± 12 pg/ml) or dexmedetomidine (50 ± 11 pg/ml); this pattern was also present at four hours (saline 130 ± 54 pg/ml; midazolam 55 ± 8 pg/ml; dexmedetomidine 62 ± 39 pg/ml) and five hours (saline 141 ± 30 pg/ml; midazolam 62 ± 20 pg/ml; dexmedetomidine 73 ± 40 pg/ml). Integrated over time revealed an area under the curve of 626 ± 137 in the saline group, 232 ± 40 in the midazolam group, and 244 ± 93 in the dexmedetomidine group. Thus the reduction in mortality effect in the sedative group was associated with a reduction in TNF-α levels in both sedated groups (Figure 2).

### Cytokine signalling: IL-6

**Figure 3:**
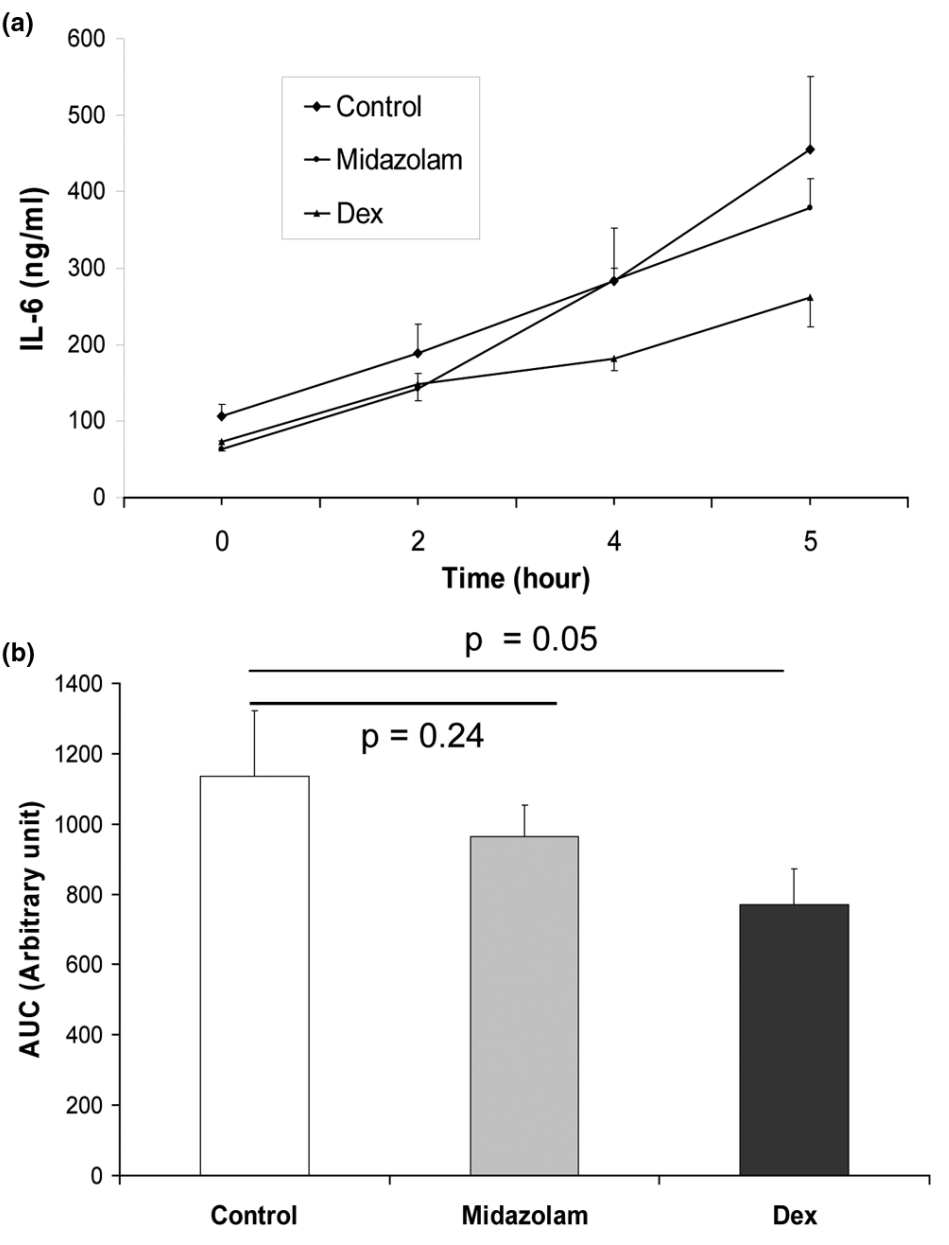
Plasma IL-6 levels immediately prior to (0 hours) and after (2, 4 and 5 hours) induction of severe sepsis by double caecal ligation and puncture in rats (n = 4 to 6).  **(a) **The actual change in levels is shown. **(b) **The total difference in levels via analysis of area under the curve (AUC) is shown. Dex = dexmedetomidine.

In contrast to sedation with midazolam, dexmedetomidine reduced IL-6 levels relative to the saline group (*P *< 0.05; Figure 3). At two hours the saline (188 ± 37 pg/ml), midazolam (176 ± 40 pg/ml), and dexmedetomidine groups were similar (50 ± 11 pg/ml). At four hours the IL-6 levels in the dexmedetomidine group (181 ± 15 pg/ml) were significantly lower than midazolam (312 ± 39 pg/ml) and saline (282 ± 70 pg/ml) groups. At six hours the IL-6 levels in the dexmedetomidine group (262 ± 38 pg/ml) were again lower than midazolam (371 ± 14 pg/ml) and saline (455 ± 96 pg/ml) groups. The mean area under the curve was 1135 ± 187 in the saline group, 1132 ± 90 in the midazolam group, and 771 ± 100 in the dexmedetomidine group.

### Effects on splenic caspase-3 expression

**Figure 4:**
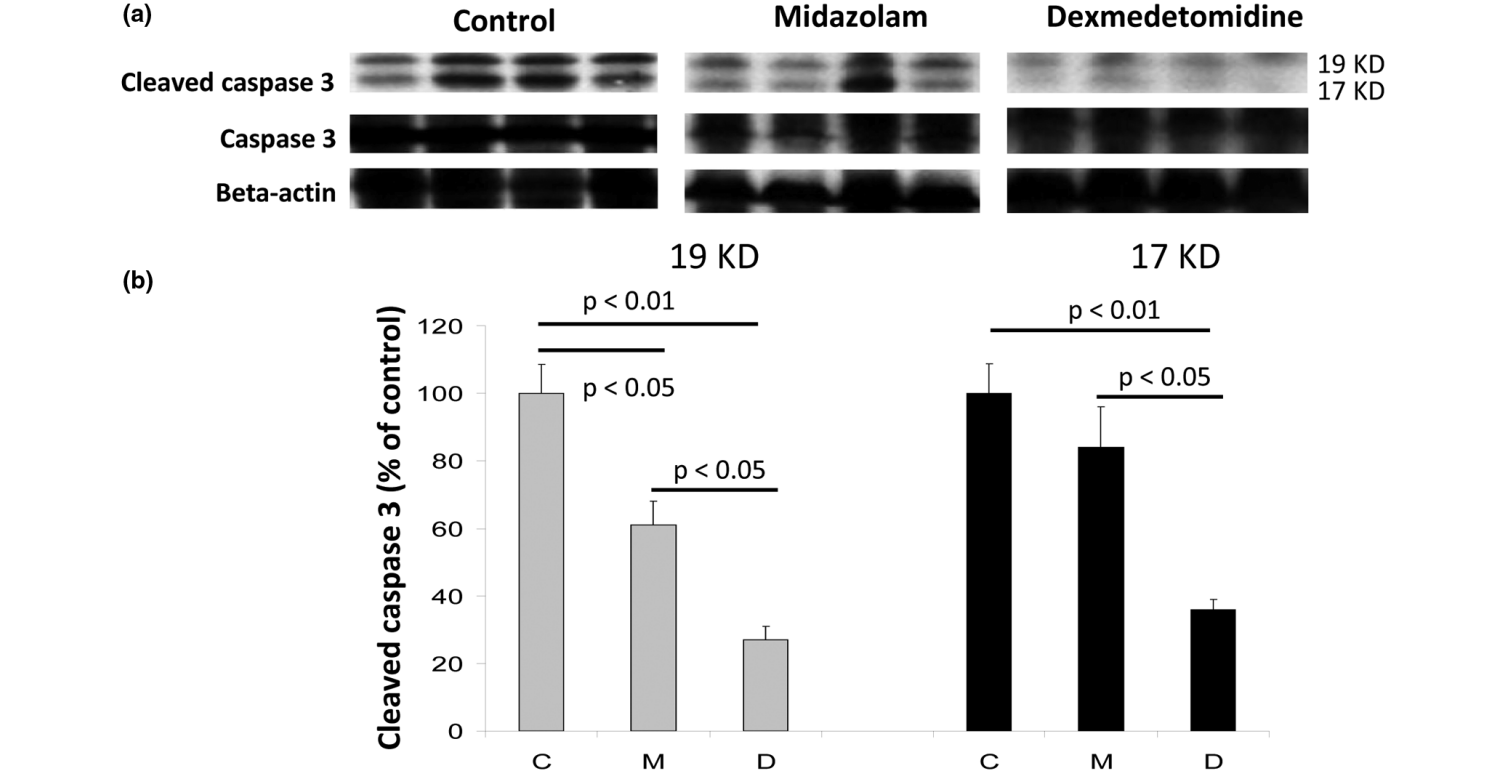
Splenic caspase-3 western blots samples from severely septic rats.  **(a) **Representative bands (each band from each one individual animal; n = 4) from the western blots are shown. **(b) **Densitometry analysis from the western blots showing quantative change in caspase-3 levels. C = control treatment (saline); D = dexmedetomidine treatment; M = midazolam treatment.

At death or at eight hours after CLIP splenic caspase-3 expression was reduced in the dexmedetomidine group relative to both midazolam and controls (*P *< 0.05; Figure 4) with similar effects on both the 17 and 19 KDa caspase-3 fractions. Interestingly midazolam reduced expression of the 17 KDa but not the 19 KDa fractions relative to saline.

## Discussion

In this model of acute, severe sepsis the sedatives, dexmedetomidine and midazolam, reduced early mortality. This mortality benefit was associated with reduced TNF-alpha signalling in both groups. Additionally, dexmedetomidine sedation also reduced IL-6 levels (*P *= 0.05) and splenic caspase-3 expression (*P *< 0.05) compared with benzodiazepine sedation. These two actions indicate that dexmedetomidine may show benefit models of sepsis explored at later time intervals.

### Caveats

This model of sepsis in healthy rats does not necessarily replicate vulnerable patients with sepsis. Although attempts were made to fluid resuscitate the animals this was in a protocol driven manner and thus was not necessarily analogous to the clinical situation where resuscitation is titrated to patient's needs determined by invasive hemodynamic monitoring. We chose not to administer antibiotics, a departure from clinical practice, because we wanted to observe the consequences of acute polymicrobial sepsis. Our model is analogous with acute sepsis that is severe enough to require provision of sedation for mechanical ventilation and can lead to death within hours in the absence of appropriate management. We chose a limited sedative period as continuous sedation cannot be provided for more than 12 hours in animals according to the institutional license and all animals received further pentobarbital boluses to allow blood sampling in the animals randomized to saline. Although we scaled the dexmedetomidine and midazolam drug doses using established methodology and there were no observable differences in the level of animal sedation, it is possible that the level of sedation did differ between the groups. Future studies looking at electroencephalogram-guided sedation are planned to overcome this caveat to our experiment. We have previously used caspase-3 expression as a marker of apoptosis for which it is well validated [2]; however, our approach of using splenic western blotting lacks specificity for vulnerable cell types such as lymphocytes, although the CLIP model does induce apoptosis in these cells. Therefore, apoptosis of other cell types (including endothelial cells and macrophages) may have contributed to the caspase-3 expression. These cells appear to have less relevance to clinical sepsis [2,3] and thus may have skewed our data.

### Sedation induced anti-inflammatory effects

Previous preclinical studies had shown that sedation with dexmedetomidine does improve mortality from endotoxic shock in rats compared with a non-sedated group [22]. Based upon the inflammatory and apoptosis biomarkers we would anticipate superior benefits of sedation with dexmedetomidine *vs *midazolam in the acute phase of sepsis; possible reasons why this putative benefit was not borne out by the mortality data may relate to the 'hyper-aggressive' septic state that appears primarily to be TNF-α dependent (as mortality benefits were associated with reduced TNF-α levels). It is noteworthy that midazolam and dexmedetomidine reduced TNF-α levels by a similar amount although previous clinical trials have suggested that dexmedetomidine was superior to midazolam in this regard [24]. Dexmedetomidine has also been shown to improve mortality and reduce inflammatory cytokine levels induced by CLIP in mice when dexmedetomidine was started prior to the sepsis [23] though the dosing schedule in this study was irregular. In our study the sedatives were commenced by infusion shortly before provoking sepsis and therefore the levels were unlikely to be therapeutic as sepsis was induced.

The anti-inflammatory effects of dexmedetomidine have now been shown against endotoxin (compared with saline) [22], in single CLIP [23], in double CLIP (compared with midazolam; Figures 2, 3) and in critically ill humans (compared with propofol [25] or midazolam [24]). How dexmedetomidine induces its anti-inflammatory effect is currently unclear though it may be related to its central sympatholytic effects [23,30] and relative stimulation of the cholinergic anti-inflammatory pathway [16,17]. Inflammation also appears to alter the effects of α_2 _adrenoceptor stimulation shifting them from a pro- to an anti-inflammatory effect [35].

The effect of the sedatives on IL-6 require further consideration as IL-6 levels are predictive of mortality in septic humans [36] and animals [37]. Therefore, the reduction of IL-6 levels by dexmedetomidine relative to midazolam and saline may prove crucial in future studies. The achieved significance value of *P *= 0.05 means the results are of borderline significance though we suspect this is due to a reduced sample size in the midazolam group. Power analysis based on our results suggests that six animals per group are required to achieve power to find a statistical difference of *P *< 0.05. Therefore our study was designed with appropriate power but a loss of two animal samples in the midazolam group, leaving a sample size of four animals in that group, may have been responsible for our result that is of borderline significance. The superiority of dexmedetomidine's ability to reduce IL-6 levels has already been shown in humans [24,25]; however, it should be noted that dexmedetomidine was administered immediately after the septic insult in this study. This is important as the timing of anti-IL-6 therapy is critical; delays greater four hours after CLIP show no benefit in septic animals [38].

How midazolam induces an anti-inflammatory effect is unclear but immune cells express both the peripheral benzodiazepine receptor [39] and gamma-amino butyric acid receptors [40] and thus at least two local targets exist for benzodiazepines. For example, midazolam suppressed lipopolysaccharide-induced TNF-α activity in macrophages, an effect that was blocked by the peripheral benzodiazepine receptor antagonist PK 11195 [39]. Midazolam also inhibits lipopolysaccharide-induced up-regulation of cyclooxygenase 2 and inducible nitric oxide synthase in a macrophage cell line. Other markers of immune cell activation (induced by lipopolysaccharide) such as IκB-α degradation, nuclear factor-κB transcriptional activity, phosphorylation of p38 mitogen-activated protein kinase and superoxide production were also suppressed by the midazolam [41].

Interestingly dexmedetomidine and midazolam appear to exert opposite effects on innate immunity. Dexmedetomidine appears to potentiate macrophage function and phagocytosis [27-29], while, as described above, midazolam inhibits it [39,41,42]. This may be related to opposing effects on p38 mitogen-activated protein kinase signaling in these cells [41,43]. Thus although both sedatives suppressed circulating cytokines, at a local level the effects on macrophages may have been very different. Benzodiazepine induced suppression of immunity has been noted against *Salmonella typhimurium *with 15 days of diazepam treatment [19] and *Klebsiella pneumoniae *with three days of diazepam treatment *in vivo *[20]. In these settings of infection, diazepam treatment increased animal mortality. Thus longer treatment times may be needed to show impairment of immune responses by midazolam than used in this study. We consider that differing effects on innate immunity may explain why critically ill patients sedated with dexmedetomidine experienced fewer infections than those patient sedated with midazolam in a recent randomized controlled trial of 366 critically ill patients [44]. Further studies addressing the relative effects of longer dosing schedules and different doses of the two sedatives on innate immune responses are in progress. It is interesting to note that daily interruption of sedative infusions appear to be associated with fewer infective complications [45]; this may be related to the reduced dose of sedatives resulting in less inhibition of the immune system. Recently, deep sedation has been associated with increased mortality in the critically ill [46] although it is unclear whether this affected immune responses. In this study we did not measure depth of sedation with electroencephalogram monitoring; however, based on recently published clinical data [46], future studies should consider this. Nonetheless our data suggests that the sedatives are equally able to reduce mortality during the acute phase of sepsis and therefore that choice of sedative in this acute phase may not matter.

### Effects of sedation on apoptosis in sepsis

Apoptotic (or programmed) cell death occurs in physiological conditions; for example, it is an important mechanism by which immune responses are controlled via activated cell death of lymphocytes. Sepsis induces apoptosis in lymphocytes, dendritic cells and enterocytes and death of these cells appear pivotal to the pathogenesis of the hypo-inflammatory phase of the condition [2,3]. Prevention of this apoptotic injury with inhibitors of the caspase enzymes [47], regarded as the final executioners in apoptosis or of over expression of anti-apoptotic proteins, has been shown to improve survival in animal models of less acute sepsis.[2,3] Critical mediators of this septic apoptotic injury include pro-apoptotic proteins such as BAX and activated caspase-3 [2,3].

Both midazolam and dexmedetomidine reduced the burden of splenic caspase-3 expression indicating that they may exert some anti-apoptotic effects in the presence of severe sepsis. It is possible that in the present model, TNF-α binding stimulated the extrinsic apoptotic cascade. Thus the observed inhibition of apoptotic markers may be, in part, due to suppression of the inflammatory response. This would account for why both sedatives showed some anti-apoptotic ability. Interestingly, midazolam was only capable of reducing the 19 KDa fragment of cleaved caspase-3; why it had such an effect is currently unclear. Nonetheless, dexmedetomidine exhibited significantly superior anti-apoptotic effects, consistent with previous reports demonstrating that dexmedetomidine could prevent apoptotic injury from hypoxia and isoflurane in neurons [26,48]. α_2 _adrenoceptor stimulation reduces pro-apoptotic proteins such as BAX and increases anti-apoptotic Bcl-2 signaling [49], indicating activity against the intrinsic apoptotic cascade. As apoptotic mechanisms are highly conserved and therefore anti-apoptotic agents are likely to work in different tissue types we hypothesized that stimulation of α_2 _adrenoceptors by dexmedetomidine may inhibit septic apoptosis. Indeed activation of AKT/protein kinase B, extracellular regulated signalling kinase and Bcl-2 improves survival in sepsis [2,3] and these effectors are upregulated by dexmedetomidine [49,50]. Therefore, the reduction in sepsis-induced splenic apoptosis is plausible (Figure 3).

The consequences of apoptosis may be more relevant in clinical sepsis and in the less acute phase of sepsis in animal models. Also, in acute severe sepsis apoptosis of cells may have a protective effect by dampening the immune response; improved mortality has been noted from endotoxic shock in animals treated with apoptotic cells [51]. This suggests a complex and dynamic set of circumstances pertain during sepsis expressed in apoptotic and inflammatory responses that are observed at different times. Indeed corticosteroids show anti-inflammatory effects (that have correlated with increased speed of reversal of septic shock in the CORTICUS trial [10]) but exacerbate lipopolysaccharide-induced apoptosis [52]. However an agent, such as dexmedetomidine, that can combat both inflammation (in the early phase of sepsis) and apoptosis (in the later phase of sepsis) could have particular utility in septic patients. These data also help explain the remarkable mortality benefit we have seen in septic patients from the MENDS study [32]. This hypothesis will need evaluation in further preclinical studies.

## Conclusions

Sedation in acute severe sepsis may be of benefit to dampen the accompanying cytokine storm and reduce mortality. Dexmedetomidine offers some theoretical advantages over midazolam that may become evident in a less severe septic model. Nonetheless, although sedation appears therapeutic in the acute phase of sepsis, choice of sedative at this stage is unlikely to determine outcome (Figure 1).

## Key messages

• Sedatives exert different immunomodulatory effects during sepsis and may improve outcome in acute severe sepsis.

• Dexmedetomidine exerts an anti-apoptotic effect in sepsis that may be of use in more chronic septic states. Further studies are required to investigate this potential benefit.

## Abbreviations

CLIP: cecal ligation and double intestinal puncture; IL: interleukin; TNF: tumour necrosis factor.

## Competing interests

MM discovered and patented the anesthetic properties of dexmedetomidine in 1987. He reverted his rights to the patent to Orion Farmos for $250,000 in support of laboratory activities. MM has received grant support, speakers fees and honoraria from Orion, Abbott Labs (who registered dexmedetomidine for its sedative use) and Hospira (who market dexmedetomidine).

## Authors' contributions

The hypothesis was developed by RDS in conjunction with MM and DM. All authors (HQ, XW, RDS, DM, and MM) contributed to the study design and interpretation. HQ and XW performed the experiments. RDS drafted the manuscript with DM and QH. All authors reviewed the manuscript and contributed to editing it for publication.
